# Evidence and evidence gaps – an introduction

**DOI:** 10.3205/cto000138

**Published:** 2016-12-15

**Authors:** Gabriele Dreier, Jan Löhler

**Affiliations:** 1 German Study Center of Oto-Rhino-Laryngology, Head and Neck Surgery, Bonn, Germany; 2 Clinical Trials Unit of the Medical Center – University of Freiburg, Germany; 3 Scientific Institute for Applied Oto-Rhino-Laryngology, Bad Bramstedt, Germany

**Keywords:** evidence-based medicine, clinical trial unit, registry, transparency, medical devices

## Abstract

**Background:** Medical treatment requires the implementation of existing evidence in the decision making process in order to be able to find the best possible diagnostic, therapeutic or prognostic measure for the individual patient based on the physician’s own expertise.

Clinical trials form the evidence base and ideally, their results are assembled, analyzed, summarized, and made available in systematic review articles. Beside planning, conducting, and evaluating clinical trials in conformity with GCP (good clinical practice), it is essential that all results of conducted studies are publicly available in order to avoid publication bias. This includes also the public registration of planned and cancelled trials.

**History:** During the last 25 years, evidence-based medicine became increasingly important in medical care and research. It is closely associated with the names of Archibald Cochrane and David Sackett. About 15 years ago, the Deutsche Cochrane Zentrum (Cochrane Germany) and the Deutsche Netzwerk Evidenzbasierte Medizin e.V. (German Network for Evidence-based Medicine, DNEbM) were founded in Germany. In the International Cochrane Collaboration, clinicians and methodologists come together on an interdisciplinary level to further develop methods of evidence-based medicine and to discuss the topics of evidence generation and processing as well as knowledge transfer.

**Problem:** Evidence is particularly important for physicians in the process of decision making, however, at the same time it is the base of a scientific proof of benefit for the patient and finally for the payers in health care. The closure of evidence gaps requires enormously high staff and financial resources, significant organizational efforts, and it is only successful when clinical and methodical expertise as well as specific knowledge in the field of clinical research are included. On the other hand, the knowledge has to be transferred into practice. For this purpose, practice guidelines, meetings, databases, information portals with processed evidence as well as specific journals and finally teaching are appropriate vehicles. One problem is the multitude of information so that knowledge gaps may affect the clinical routine despite actually existing evidence. Generally, it still takes several years until new knowledge is implemented in daily routine.

**Tasks:** The German Society of Oto-Rhino-Laryngology, Head and Neck Surgery (Deutsche Gesellschaft für Hals-, Nasen- und Ohren-Heilkunde, Kopf- und Hals-Chirurgie e.V., DGHNOKHC) and the Professional Association of Otolaryngologists (Deutscher Berufsverband der HNO-Ärzte e.V., BVHNO) have fundamental interest in supporting their members in generating, processing, and providing evidence as well as accompanying knowledge transfer. It encompasses the fields of diagnostics, therapy, and prognosis in the same way as prevention and applies to medicinal products as well as to medical devices or surgical procedures. The base for this is the regular assessment of evidence gaps, also in the area of established procedures, that has to be followed by a prioritization of research questions and the subsequent initiation of clinical research. In addition, large trials verifying therapies and diagnostics, for example in the context of daily conditions after approval, can only be conducted combining all resources in the ENT community.

**Method, results, and outlook:** Together, the executive committees of the DGHNOKHC and the BVHNO founded the German Study Center of Oto-Rhino-Laryngology, Head and Neck Surgery (Deutsches Studienzentrum für Hals-, Nasen- und Ohren-Heilkunde, Kopf- und Hals-Chirurgie, DSZ-HNO). First projects have been initiated, among those a clinical trial on the therapy of sudden hearing loss supported by the BMBF and a survey on evidence gaps in oto-rhino-laryngology. It seems to be both reasonable and feasible to make available methodological expertise via such an infrastructure of a study center for physicians in hospitals and private practices in order to support clinical research and to implement the principles of evidence-based medicine in daily routine.

## 1 Evidence-based medicine and transparency in the focus of interest

In accordance with the motto of the 87^th^ Annual Meeting of the German ENT Society, this journal volume contains articles on evidence and evidence gaps in oto-rhino-laryngology. It is certainly unique to focus on the lack of knowledge and missing research, besides presenting new knowledge, results of basic and clinical research. At the same time, these journal articles concentrate on a discipline, the evidence-based medicine, that has indeed reached medical care but is not yet “at home” there, as it was described by Baethge in an article in the Deutsche Ärzteblatt [1]. Yet, evidence-based medicine is not a novel young discipline, physicians of all medical fields daily act in an evidence-based way.

One important impediment to the successful implementation of evidence-based medicine is the physicians’ reservations. But evidence-based medicine is neither a “cookbook medicine” dictating medical care, nor is it a means to save money or to reduce medical care options for the patients or to minimize the refunding of the service providers.

Another problem is the lack of resources and structures especially in Germany while in Anglo-Saxon and Scandinavian countries as well as in the USA and Canada institutes were established and programs were initiated to generate, process and provide knowledge [2]. In order to allow evidence-based medical care, first of all time and money is needed. Existing evidence has to be collected, included in a meta-analysis if necessary, evaluated, and made available and it has to be found and applied by treating physicians. Even just the retrieval of literature is sometimes difficult because of cost-intensive and limited access to databases, libraries, and publications. When existing evidence is not processed in the sense of regularly actualized guidelines or systematic review articles, the individual physicians are forced to collect the existing evidence from different sources and analyze it themselves. But often they do not have the time beside their work in clinical practice and research.

## 2 Evidence-based medicine – what, how, when, why?

### 2.1 Historic development, pioneers, and the work of the international Cochrane Collaboration

Even though physicians have been looking for a basis for their medical action for more than 200 years now by performing trials and evaluations, and James Lind’s publication of the efficacy of vitamin C for scurvy in 1753 is regarded as being the first clinical trial [3], the current international discipline of evidence-based medicine is clearly associated with the general reflections of Archibald Cochrane.

Cochrane who was a British physician and epidemiologist, focused on randomized clinical trials and healthcare research. In 1972, he published an article entitled “Effectiveness and Efficiency. Random reflections of health services”, which was the cornerstone for the following international development of evidence-based medicine and the foundation of the Cochrane Collaboration, which is named after him [4]. Already at that time, Cochrane requested among others the application of unique quality criteria for the assessment of published research and transparency created by public study registers. Both requirements have not lost their relevance since.

The International Cochrane Collaboration gathers methodologists and clinicians on a global level in order to for example further develop methods and generate and process knowledge on an interdisciplinary scale. In every medical discipline, there are different groups that deal with single indications and issues. In the Cochrane Ear, Nose- and Throat Disorders Group for example, methodological scientists and physicians from the United Kingdom, the Netherlands, Australia, Canada, and the USA and other countries work together [5]. The authors of the review articles as well as scientific consultants, statisticians, (medical) experts etc. working with the Cochrane ENT Disorders Group however, come from different countries all over the world, and new physicians and scientists as well as representatives of health care professions and patient groups who want to contribute to generating and processing evidence are always welcome.

At the occasion of the Annual Meeting of the German ENT Society in Dortmund in 2014 Martin Burton, who is one of two coordinating editors of the Cochrane ENT Disorders 

Group – together with Anne Schilder from Utrecht – and at the same time director of the UK Cochrane Centre, stated that the foundation of the German Study Center of Oto-Rhino-Laryngology, Head and Neck Surgery was an important milestone supporting physicians in planning and performing clinical trials and systematic reviews.

Co-founder of the international Cochrane Collaboration was David Sackett, a physician and researcher who died in May 2015. He is considered a pioneer of clinical epidemiology and evidence-based medicine. Already in 1967, he established a professorship for clinical epidemiology and biostatistics at the McMaster University of Hamilton, Canada, which was an international novelty. Sackett simultaneously worked as physician, conducted randomized clinical trials, refined the methods of clinical research, and in addition he introduced the results of his work in teaching and education. In 1996, he wrote an editorial entitled “Evidence based medicine: What it is and what it isn’t”, which in its modified form provides a definition and delimitation of evidence-based medicine that is still valid today [6]. One of Sackett’s former students and later co-worker at the McMaster University of Hamilton was Gordon Guyatt who was the first to use the term “evidence-based medicine” in an article published in 1991.

In 1993, The *Journal of the American Medical Association* (JAMA) published a series entitled “The Users’ Guide to the Medical Literature” that was summarized as a book with the same title in 2002 and (co-)editored by Gordon Guyatt. In January 2015, the third edition was issued explaining the principles and methods of evidence-based medicine and their application in comparably new fields such as stratified medicine/precision medicine [7]. In accordance to the title, support is given how readers may interpret and assess publications and also how the relevant literature is found. Because of the enormously increasing number of articles, the user is not only referred to literature databases, but also to databases containing processed evidence such as systematic review articles or practice guidelines.

In 1998, the informal working group of the “Deutsches Netzwerk Evidenzbasierte Medizin” (DNEbM, German network of evidence-based medicine) was founded in Berlin, Germany. In 2000, the foundation of the association with the same name followed [8]. The German Cochrane Centre (Deutsches Cochrane Zentrum, DCZ, now called Cochrane Germany) was founded in 1999 at the Medical Center, University of Freiburg, Germany. Until 2013, the DCZ was part of the Department of Medical Biometry and Medical Informatics; since 2014, Cochrane Germany is an independent department of the Medical Center – University of Freiburg and it is financed by the Medical Center and the German Ministry of Health [9].

### 2.2 Evidence-based medicine in practice

According to the current definition, evidence-based medicine means that the treating physician takes existing external evidence, his own experience and expertise and the wishes of the individual patient he is treating into account in order to find an appropriate treatment for the patient. In this context, not only the medical-clinical situation of the patient is taken into account, but also his individual preferences are considered. Evidence-based medicine can only be applied based on external data, medical knowledge and experience, and the patient’s circumstances.

However, evidence-based medicine should not only support therapeutic decision making but also encompass prognosis, prevention, and diagnosis. Of course it is desirable not only to investigate new procedures and therapies, but also established methods and treatment procedures. Especially in the surgical field it is possible that traditions or regional schools of thought continue adopting procedures without questioning them. Finally, therapies might be effective – but perhaps not always and not for each patient. Also in this context, evidence-based medicine may be helpful to provide a base for decisions and to distinguish between patients who might benefit from a treatment – pharmaceutical or surgical – and those who are probably non-responders. Especially in cases of interdisciplinary procedures such as the combination of radiotherapy and surgery or surgery and chemotherapy or different surgical techniques with application of different medical devices (e.g. laser or devices assisting certain surgical techniques) a sound scientific data collection and clinical research are necessary. The focus may also be placed on type, time, or chronological order of the combinations.

Also the service of health care professionals such as nursing staff or other therapeutic professions like physiotherapists, speech therapists, or technical professions such as hearing care professionals can and should be based on principles of evidence-based medicine. In these fields clinical research and generation and transfer of new knowledge are as important as in classical medical professions.

### 2.3 Clinical trials – the base of evidence-based medicine

In this context, randomized, controlled clinical trials (RCTs) are the acknowledged gold standard, but also other types of studies, e.g. large-scale cross-sectional and cohort studies, are essential regarding questions on prevention and prognosis. Furthermore, after proof of efficacy, new therapeutic concepts should be verified by large trials under everyday conditions. Mostly, the initiative for those post-marketing trials has to originate from non-commercial, academic/clinical groups because after approval the interest of the industry is generally rather low to conduct cost-intensive studies on medicinal products or medical devices. This makes it even more important to create structures allowing to conduct high-quality trials and also to provide sufficient public means to finance those trials. So-called non-inferiority trials become more and more important because many new procedures and therapies are not necessarily more effective, but the same efficacy is associated with e.g. less undesired (side) effects or the application is easier (e.g. cooling for storage is not necessary, oral instead of intravenous administration, less frequent application).

Often the idea of such an improved therapy arises in the clinic, ideally there is even a cycle of (basic) research, transfer into the clinic, and further research.

### 2.4 Evidence-based medicine and clinical trials in ENT – particularities and opportunities

In otolaryngology, surgical procedures as well as the use of medical devices play an important role in diagnostics and therapy. In addition there is the interdisciplinary treatment for example of oncologic patients in cooperation with other disciplines such as radiotherapy or palliative care.

Trials in this setting have specific methodological and regulatory characteristics compared for example to trials on pharmaceuticals. 

#### 2.4.1 Clinical trials with medical devices

Regarding pharmaceutical products, clinical trials are the basic precondition for approval. Since the introduction of the Arzneimittelmarktneuordnungsgesetz (AMNOG, German Pharmaceutical Market Reorganization Act) in January 2011 in Germany, new medicinal products are evaluated with regard to their additional benefit in comparison to existing pharmaceuticals [10]. In this context, especially the patient-oriented benefit plays a significant role. This means that it is not enough to prove an effect on surrogate parameters such as for example the reduction of hypertension, but trial endpoints must reflect a real benefit for the patient, e.g. the reduced occurrence of strokes. Furthermore, the improvement of the handling of the medicinal product, thereby increasing compliance, may be significant or the reduction of undesired effects in comparison to existing pharmaceuticals. The pharmaceutical company is obliged to submit an extensive dossier encompassing among others the complete data of all trials conducted. The result of the early benefit assessment determines the price for the pharmaceutical product to be achieved. The Gemeinsame Bundesausschuss (G-BA, Federal Joint Committee) asks the Institut für Qualität und Wirtschaftlichkeit im Gesundheitswesen (IQWiG, German Institute for Quality and Efficiency in Health Care) to assess the benefit [11]. Regarding medical devices, however, an approval is not obligatorily related to conducting clinical studies or providing proof of benefit, except for special medical devices of the highest risk class. A clinical assessment is sufficient in most of the cases and the manufacturer may refer to comparative data of other medical devices. By issuing Article 10 § 2 and the new attachment 3 of the Medizinproduktebetreiberverordnung (MPBetreibV, Medical Devices Operator Ordinance) in 2014, the legislator has created some new obligations for certain implantable medical devices, e.g. extended obligations regarding information and documentation for users/hospitals that came into force in October 2015 after a transitional period [12], however, the obligation of the manufacturer to conduct clinical trials and to provide evidence for clinical benefit is still missing, which is also criticized by the IQWiG in its annual report of 2014 [13]. In 2012, the EU-wide process was started to revise the existing regulations on medical devices [14]. The objective was to ensure:

- a consistently high level of health and safety protection for EU citizens using these products;
- the free and fair trade of the products throughout the EU;
- that EU legislation is adapted to the significant technological and scientific progress in this sector over the last 20 years [15].

In addition, the request of evidence is explicitly stated:

“Revisions included the extending of the scope for legislation; better supervision of independent assessment bodies; clear rights for manufacturers/distributors; and stronger requirements for medical evidence.”

The status of EU legislation and the adoption of the “Proposal for a REGULATION OF THE EUROPEAN PARLIAMENT AND OF THE COUNCIL on medical devices, and amending Directive 2001/83/EC, Regulation (EC) No 178/2002 and Regulation (EC) No 1223/2009” can be retrieved on the according website.

The problem of a missing obligatory proof of benefit is that neither the physician nor the patient can include such a proof of benefit in the decision making process. If researchers and clinicians want to act in the sense of evidence-based medicine, they have to plan, organize, and conduct suitable trials at their own expenses because the manufacturer is not interested as long as he gets the market approval even without such a proof of benefit. However, this is also a disadvantage for the industry when the manufacturer cannot achieve a reimbursement by the health insurance companies due to the institutions deciding on reimbursement requiring a proof of benefit. Possibly effective and beneficial medical devices may therefore not be used because neither the patient nor the service provider is able to or willing to bear the costs. In some cases, a product is implemented in the service catalogue but the refunding is disproportionate to the costs the user has to pay. The use of some products causes current expenses which may lead to the situation that hospitals get for example a device at a low price but have to bear the operating expenses. This is comparable to the obligation to buy expensive cartridges for cheap printers. At this point (if not before) clinical studies on the benefit become necessary that should be requested reasonably at the time of approval. On the other hand, clinicians and in particular also manufacturers have data at their disposal, e.g. from documentations for quality assessment, that are not systematically analyzed. The IQWiG discussed also this issue in its annual report of 2014 [13].

It would be reasonable in the sense of evidence-based medicine to request a proof of benefit with focus on patient-oriented endpoints including all existing data, to conduct clinical trials, and to publish the results in publicly available registers, as it is common practice with pharmaceutical products. Such a procedure could significantly facilitate several controversial questions, especially in the context of reimbursement. Meanwhile the requirements of trials for medical devices are similar to those of pharmaceutical products so that sufficient expertise for supporting the conduct of such trials is nationally available, e.g. provided by the network of coordination centers for clinical trials (KKSN) [16]. The Clinical Trials Unit of the Medical Center – University of Freiburg which is providing expertise for the German Study Center of Oto-Rhino-Laryngology, Head and Neck Surgery (Deutsches Studienzentrum für Hals-, Nasen- und Ohren-Heilkunde, Kopf- und Hals-Chirurgie, DSZ-HNO) is also founding member of the KKSN network [17].

#### 2.4.2 Clinical Trials in surgery

Another characteristic of clinical research in ENT and Head and Neck Surgery is the necessity to evaluate also surgical procedures in controlled clinical trials with regard to their benefit. But other surgical disciplines already managed to prove that this is possible and that it is feasible to conduct also randomized, controlled and (partly) blinded trials. The study center of the German Society of Surgery (Deutsche Gesellschaft für Chirurgie, SDGC) in Heidelberg, which is also (extraordinary) member of the network of coordination centers for clinical trials, represents a center that is committed to evidence-based medicine in surgery [18]. The German ENT Study Center considers it useful to benefit from synergies, as it can be seen for example when conducting courses for principal investigators that also focus on the special features of surgical trials. Often missing qualifications/(principal) investigator certificates are a reason even for interested clinicians not to plan and conduct clinical trials.

### 2.5 Evidence and evidence gaps – a hot topic for scientific societies and professional associations

The identification and filling of evidence gaps is a central interest of a scientific society and a professional association in order to further develop the discipline, transfer the knowledge and to assure evidence-based diagnostics and therapy for the patients independently from their place of residence. Evidence generation is last but not least the base for patients to access procedures/therapies/medical devices and services free of charge and for the association’s and society’s members to get reimbursed.

Exactly the networks that may be reached by a scientific society and a professional association are the basis to identify evidence gaps (see the article by Löhler et al. in this issue [19]) and to conduct clinical trials as well as to support knowledge transfer. Randomized controlled trials as well as large cohort studies need the cooperation of many investigators to include different patients that are seen either in practices or at (university) hospitals. Furthermore it is easier to implement the results in the daily routine if many of the later users are involved in the trials, which could already be shown in other contexts [20].

Additionally it is reasonable to act on a long-term basis and to include the results of clinical research not only regularly in practice guidelines but also to systematically update those guidelines and to provide access to (processed) evidence. Finally, this will help physicians in their daily practice to make evidence-based decisions. During the last years, the GRADE procedure was implemented for writing practice guidelines [21]. In this context, an even weak evidence may lead to a recommendation when the benefit is potentially high, e.g. in cases of life-threatening infections where no approved therapy but only supportive treatment is available. Here, even therapies with only weak benefit could be recommended. Often, also preferences of the users play an important role. Even if the potential risk for embryonic damage is rather low, pregnant women would decide more often in favor of a painful and cost-intensive therapy than non-pregnant patients with the same indication. Differentiated evidence-based guidelines are required. Unfortunately, the writing of guidelines is not sufficiently acknowledged in the academic world; in general, incentives to contribute to clinical trials are poor for physicians in academic institutions as well as for physicians in hospitals and private practices. Structures have to be created or existing ones have to be used to facilitate external fund raising, reduce additional work load, and to do the administrative, regulative, and coordinating work for the researching physicians. The foundation of the DSZ-HNO can be considered as a step into the right direction.

Another topic in the context of evidence-based medicine is the verification of the common practice. The question must be asked if sometimes measures are taken despite existing and known evidence that they do not have a benefit in the specific situation but they are taken nonetheless “for safety reasons” or because the patient wants them or in order to be able to offer at least something. In the last years the initiative called “choosing wisely” was founded. However, because of the special situation of the different health care systems in the USA and in Germany it cannot be easily transferred. The results of some workshops on that topic can be found for example on the website of the DNEbM [8]. In 2014, Friederike Klein published some examples of not necessarily required measures in ENT that are taken from http://www.choosingwisely.org/ [22].

## 3 Support of evidence-based medicine in oto-rhino-laryngology, head and neck surgery: the foundation of the German Study Center was a milestone

On the one hand, the field of oto-rhino-laryngology, head and neck surgery is under-represented in the context of the support provided by the grant program “Clinical Trials” of the German Research Foundation (Deutsche Forschungsgemeinschaft, DFG) and the Federal Ministry of Education and Research (Bundesministerium für Bildung und Forschung, BMBF) [23], on the other hand only few ENT funding applications are submitted. It can only be speculated on whether this is due to the characteristics of the clinical questions that refer rather to medical devices and surgical procedures than to classical trials on medicinal products and thus potential applicants possibly lacking the specific expertise and support, or whether it is because clinical trials are less established in the daily routine of clinics and practice also because the pharmaceutical industry conducts fewer studies in ENT compared to for example in the field of internal medicine.

In order to promote clinical research, the German Society of Oto-Rhino-Laryngology, Head and Neck Surgery and the German Professional Association of ENT Specialists founded the German Study Center of Oto-Rhino-Laryngology, Head and Neck Surgery (DSZ-HNO) in 2013. They deliberately avoided establishing new structures. The center is associated with the Clinical Trials Unit of the Medical Center – University of Freiburg and uses the already existing expertise for example in the fields of biometry, study assistance, project coordination, regulation, cost calculation, and data management. Furthermore, there is a close cooperation between the Clinical Trials Unit of the Medical Center and the German Cochrane Center that is also located in Freiburg. In this way a special relation to evidence-based medicine is assured and scientific expertise can be used.

The aim of the two institutions – medical society and professional association – is to support their respective members not only in implementing the best available evidence in their medical care but also in identifying evidence gaps together, ideally also in closing the gaps, and to processing the new knowledge so that it may be included in daily practice and routine. In this context it is important that both founding institutions guarantee the study units’ independency. Current and future projects are financed by public funds. Of course this does not exclude conducting clinical trials/cooperation with the industry if it seems to be reasonable or essential, e.g. for marketing approval trials or when data is used that must be provided by the manufacturer.

### 3.1 Evidence from practice into practice – the idea of the scientific society and the professional association of identifying, prioritizing and ideally filling evidence gaps together – first steps

Since its foundation in 2013, the DSZ-HNO did not only provide advice for numerous investigators but also help initiate trials from different fields of oto-rhino-laryngology, head and neck surgery. The variety of the projects reflects the broad spectrum of the discipline. Surgical procedures as well as trials on medical devices and pharmaceutical products have been supported discussed and initiated. External funding by for example the BMBF could be raised.

In order to identify evidence gaps where they become primarily obvious via a systematic approach in the sense of a circle from daily practice back into practice, a survey was carried out among physicians working in private practice and those working in hospitals in 2015 (see article by Löhler et al. [19]). In this context it was essential that the cooperation of the scientific society and the professional association contributed to the fact that all members of both institutions could be contacted. Due to the specific support by the DSZ-HNO it was possible to benefit from the expertise of the clinical trials unit of the Medical Center – University of Freiburg, where methodologically working scientists like statisticians cooperate with physicians, study assistants, data managers or project coordinators, and the close relationship to Cochrane Germany allows access to professional expertise on evidence-based medicine.

Furthermore it is a challenge in such a project to differentiate between real evidence gaps and knowledge gaps which can be observed because of the problem of knowledge transfer which is not satisfactorily resolved in any medical discipline on neither a national nor an international scale.

Instead of writing a systematic review article on each single clinical question, it seems to be more feasible on the one hand to first of all establish different decentralized groups that are supported centrally, or to join for example Cochrane Groups, or to even found new ones as so-called “editors”. Up to now only two Cochrane Groups have their base in Germany. On the other hand it may be reasonable to search for existing evidence and summarize it in a table first before writing a systematic review; this type of evidence mapping will be described in the article by Löhler et al. [19].

The search for existing evidence and the subsequent evaluation is certainly a challenge not only because of the enormous number of publications. Different pitfalls may lead to biased results and thus to potentially false conclusions. One problem is for example that only about 50% of all trial results are published which leads to the so-called publication bias. In some cases, literature is not even found because it is not indexed in large databases or because the articles are written in a foreign language.

In order to at least counteract the publication bias, the DSZ-HNO implemented an ENT- specific study sub-register that is managed in cooperation with the national study register acknowledged by the WHO: the German Register for Clinical Trials (Deutsches Register Klinischer Studien, DRKS). This register is visible on a national as well as an international level [24], [25].

The further processing of existing evidence, the prioritization of research questions and the subsequent closing of evidence gaps require a long and stable cooperation of all disciplines and staff in hospitals and private practices so that this knowledge is implemented in clinical routine afterwards. Missing knowledge and missing evidence are harmful for patients in the same way as missing therapeutic options when risk and benefit cannot be estimated reliably. Patients can only come to an informed decision if they know the existing evidence and in single cases might for example even prefer a higher quality of life instead of a marginal prolonged life.

Trials on patient-oriented endpoints are essential and the contact to patients and clinicians represents the basic condition for clinical research. In this context, the structure of the DSZ-HNO under the guidance of the scientific society and the professional association offers the best conditions. With coordinated, evidence-based research including all sectors, a contribution can be made in the sense of the Lancet series entitled “Research: Increasing Value, Reducing Waste” [26]. The whole series can be retrieved under [27]. Consequently the project of the DSZ-HNO was invited to the REWARD/EQUATOR Meeting in Edinburgh in September 2015 (REWARD: Reduce Research Waste and Reward Diligence; EQUATOR: Enhancing the Quality and Transparency of Health Research) [28].

## Notes

### Competing interests

The authors declare that they have no competing interests.
